# A Method for Visualization of Fine Retinal Vascular Pulsation Using Nonmydriatic Fundus Camera Synchronized with Electrocardiogram

**DOI:** 10.1155/2013/865834

**Published:** 2013-03-10

**Authors:** Dinesh Kant Kumar, Behzad Aliahmad, Hao Hao, Mohd Zulfaezal Che Azemin, Ryo Kawasaki

**Affiliations:** ^1^School of Electrical and Computer Engineering, RMIT University, 124 Latrobe Street, Melbourne, VIC 3000, Australia; ^2^Department of Allied Health Sciences, International Islamic University Malaysia, 25200 Kuantan, Pahang, Malaysia; ^3^Department of Public Health, Faculty of Medicine, Yamagata University, Yamagata 990-9585, Japan

## Abstract

Pulsatile changes in retinal vascular geometry over the cardiac cycle have clinical implication for diagnosis of ocular and systemic vascular diseases. In this study, we report a Vesselness Mapping of Retinal Image Sequence (VMRS) methodology to visualize the vessel pulsation and quantify the pulsatile motions in the cardiac cycle. Retinal images were recorded in an image sequence corresponding to 8 segments of the cardiac cycle using a nonmydriatic fundus camera (Canon CR45, Canon Inc., Japan) modified with ECG-synchronization. Individual cross-sectional vessel diameters were measured separately and the significance of the variations was tested statistically by repeated measures analysis of variance (ANOVA). The graders observed an improved quality of vessel pulsation on a wide region around the optic disk using the VMRS. Individual cross- sectional vessel diameter measurement after visualization of pulsatile motions resulted in the detection of more significant diameter change for both arterioles (3.3 *μ*m, *P* = 0.001) and venules (6.6 *μ*m, *P* < 0.001) compared to individual measurement without visualization of the pulsatile motions (all *P* values > 0.05), showing an increase of 2.1 *μ*m and 4.7 *μ*m for arterioles and venules, respectively.

## 1. Introduction

Retinal imaging has enabled direct and *in vivo* assessment of human's body circulation system and is applied for the detection of major systemic vascular diseases, including ischemia [1], coronary heart diseases [2] and diabetes mellitus [3] and its complications [4–6]. A number of studies [7, 8] have also reported the clinical application of dynamic retinal image processing for the investigation of pulsatile properties influenced by cardiac rhythm over time. This pulsatility is expected as a result of change in blood volumetric flow entering the ophthalmic vascular system under certain level of intraocular pressure during the peak systolic and diastolic phases of cardiac cycle, which can serve as a potential feature to rule out some clinical signs.

An example of pulsatile property observable from the retina is the spontaneous venous pulsation (SVP), which is available in approximately 90% of the patients [9, 10]. It is caused by the variation in the pressure gradient between the intraocular retinal veins and the retrolaminar portion of the central retinal vein (CRV) [11], visible as rhythmic changes the in diameter of one or more veins near or on the optic nerve head. Its clinical relevance is for differentiating early papilledema from pseudopapilledema, detection of elevated intracranial pressure (≥14 mmHg), and other pathological conditions [8]. In addition to SVP, pulsation of veins outside the optic disk (OD), such as the serpentine movement of principal arteries, pulsatile motion of small arterioles, and movement of optic nerve head are other features that can be visualized with the help of dynamic fundoscopy [8]. 

The need for dynamic assessment of the changes in retinal microcirculatory system has led to the development of Dynamic Vessel Analyser (DVA) [12] which is able to study the blood flow in the retinal vasculature and observe the vessel diameters as a function of time in a live video. It provides higher sampling rates (>20 frames per second) and high resolution, but it can observe only limited segment of individual arteriole and venule, and the methodology is suitable for highly specialised facilities. It has been observed that not all the vessels show sign of pulsation [7, 8] which indicates that a narrow visual field device (30 degrees) such as the DVA can miss some vessels segments with pulsatile features. Furthermore, DVA requires pupil dilation and flickering light stimulation which may influence the eye vasculature resulting in incorrect observations [13]. The reliability and reproducibility of flicker responses is questionable and there are questions regarding possible increase in the retinal vessel diameter, retinal blood flow, and optic nerve head blood flow in response to the flicker stimulation [13].

The alternative to the DVA is to modify the static fundus camera such that it can be used for the assessment of dynamic changes. Chen et al. [7] used monochromic red-free photographs taken with a fundus camera synchronized with electrocardiogram (ECG) and suffered from random selection of the points showing pulsatile features. Moret et al. [8] applied principal component analysis (PCA) to an image sequence acquired by a confocal scanning laser ophthalmoscope (CSLO) equipped with near infrared CCD camera to capture video. However, as no synchronization was performed with ECG, the movies turned out to start and stop at different phases with respect to cardiac cycle and required manual trimming to avoid discontinuities when looping. 

In this study, we have proposed a methodology for effective visualization of temporal changes in retinal vascular geometry during normal cardiac rhythm and studied the importance of premeasurement visualization in the quantification of retinal vessel pulsations. Unlike DVA that uses flickering light, the proposed method uses a single-flash operating (red free visible light) nonmydriatic digital eye fundus camera and allows for obtaining images with larger visual field (45 degrees). It has been hypothesized that visualization of pulsatile features prior to the diameter measurement can lead to better identification of the pulsatile vessels. This will improve the ability of the examiner to observe the vessels and identify all the pulsatile vessels and can help reduce the measurement errors due to random selection of vessel segments and increase significance level of the overall diameter change during trend estimation. The proposed technique is based on vesselness mapping of each image in a sequence of retinal images obtained using a modified fundus camera with ECG synchronization [14]. Each of the retinal images in the sequence is transformed using the gradient magnitude of hessian eigenvectors orientation matrix and masked to remove the distracting artifacts close to the vessel boundaries to obtain the vesselness map image sequence. The proposed method is called Vesselness Mapping of Retinal Image Sequence (VMRS) and enables the visualization of temporal microvascular pulsations on both main vessels and corresponding branches providing a framework for further analysis and vessel caliber measurement.

## 2. Materials and Methods

### 2.1. Subjects

Twelve healthy volunteers (9 men and 3 women) with average age of 36 (ranged from 21 to 56) participated in this study. The participants were made to rest for 15 minutes prior to the experiment to ensure stable haemodynamic condition. After the participants were rested, their blood pressure and pulse rate were recorded using a digital sphygmomanometer. The mean (SD) systolic and diastolic pressures of all the participants were obtained: 124.27 (8.85) and 73.63 (7.31) mmHg, respectively, with pulse (per minute) rate of 73.36 (11.26). The study was approved by the RMIT Research Ethics Committee and conducted in accordance with the Declaration of Helsinki of 1975, as revised in 2004. The exclusion criteria were (i) prescribed or other medication on the day of the experiment, (ii) hypertension, (iii) history of cardiovascular disease, (iv) diabetes, (v) eye disease, and (vi) history of eye surgery. The participants were explained the project purpose, procedures, risks, discomforts, and the duration of the test. This was done written and orally. The issues regarding confidentiality and privacy of the data, and their rights, especially regarding stopping the experiment without notice or explanations, were also discussed. Each participant signed the informed consent form prior to the experiment where they authorized the investigators to record their retina image and use these images for research and publication purposes without requiring any further permission from the participants. 

### 2.2. Image Acquisition Technique

A standard three lead ECG monitoring system was used to monitor the heart rate. The ECG signal was preprocessed for noise reduction and for real-time detection of the R-R intervals. The ECG monitoring system was electronically connected with the trigger mechanism of a Cannon CR-1 non-mydriatic digital fundus camera equipped with a 15.1 mega pixel Cannon EOS 50D. The system was designed to generate an adjustable trigger pulse at a designated point with respect to the R peak. The shutter delays were tested and found to be consistent and thus were ignored, as this was very small compared with the R-R interval. The ECG monitoring system monitored the R-R interval and triggered an alarm for the examiner if the interval changed more than 10% from the start because this would lead to erroneous trigger points. 

**Figure 1:**
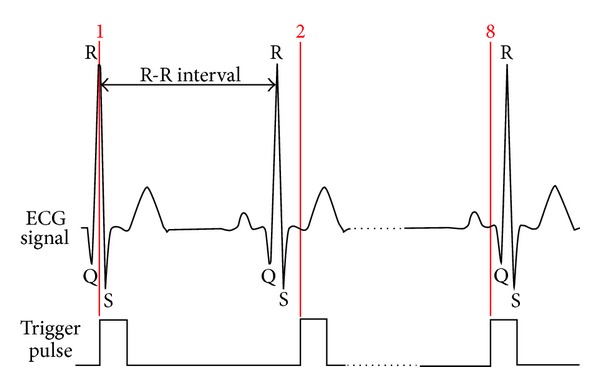
Eight distinct points with respect to QRS peak where the images were captured. Illustration of the trigger pulse and ECG signal at points 1, 2 (1/8 R-R interval time delay), and 8 (7/8 R-R interval time delay).

In this study, the R-R interval was divided into eight identical subintervals and a time-delay triggering switch triggered the shutter at nine equally spaced points (Figure 1). The cycle was divided into eight segments based on the works reported by Chen et al. [7], Moret et al. [8], and Hao et al. [15]. However, the proposed technique allows for the sampling rate to be changed to any reasonable value and may be used at a higher rate. The first image was taken at R peak, the 2nd was captured after a delay of 1/8 R-R interval, while the 8th photograph coincided with the 7/8 R-R interval. All the images were optic disc centered, in RGB, format and of size 4752 × 3168 pixels.

### 2.3. Image Alignment

During the experiment, there can be some movement of the eye leading to a relative displacement of the retinal vessels between different images. Preliminary experiments showed that this movement was comparable with the caliber pulsation and this would result in error when determining the movement due to pulsation. To remove this error, the images have to be aligned which will generate retinal image sequence free of eye movement. This will improve the visibility of pulsatile motion in retinal vasculature. 

**Figure 2:**
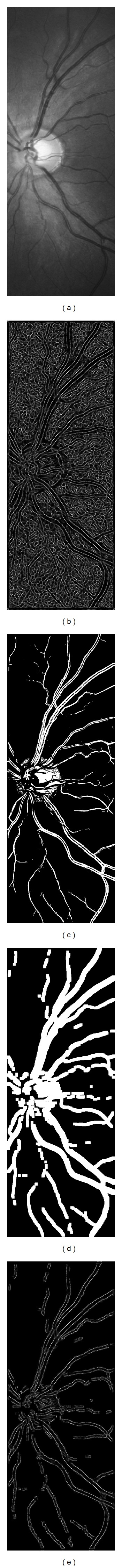
Presentation of processing outcomes at each step before image sequence generation for subject no. 11. (a) Original image (the green channel), (b) vesselness image map obtained by scale space analysis, (c) result of 2D Gabor filtering and image segmentation, (d) the mask generated by a set of morphological operations (opening followed by five consecutive dilations), and (e) the masked vesselness image with highlighted boundaries and the main blood vessels.

Image alignment was performed using i2k Align Retina software (i2k Retina, DualAlign) based on Dual-Bootstrap algorithm [16] to correct for the translational and rotational eye drift and ensure any measurement to be performed at the same spatial location. To save the computation time, the images were downsampled by a factor of two before they were fed into the registration software. Due to providing better contrast visibility [17], only the green channel was used in the alignment process. The aligned images were cropped to 576 × 1274 pixels, centering around OD which was found to be sufficient to observe all the pulsatile effects in the retinal images of the twelve subjects (Figure 2(a)).

### 2.4. Filtering and Edge Enhancement

Images may be corrupted by noise during acquisition due to numerous reasons such as the camera hardware, examiner error (e.g., focusing), and illuminations issues [18]. Traditional filtering and noise reduction methods such as Gaussian low-pass filtering use weighted average of pixel values in the neighborhood and consider the spatial variation to be slow [19]. This is based on the assumption that the neighborhood pixels have similar intensity values, and, therefore, it is appropriate to average the pixels within a small neighborhood. However, this assumption is incorrect at the edges and the low-pass filtering results in blurring the edges. This blurring is not acceptable because this would reduce the contrast at the vessel boundaries and impairs the visualization of pulsation. The bilateral filter proposed by Tomasi and Manduchi [19] has been shown to overcome this problem and was used to reduce noise without sacrificing the contrast. This is a nonlinear filter that averages the intensity values using a set of weights, which are inversely related to the intensity similarities. For mathematical formulation and details of this filter, please see Tomasi and Manduchi [19].

### 2.5. Extraction of Vesselness Map

Initial segmentation is required to build a vesselness map corresponding to edges. The boundaries were enhanced by the scale-space analysis [20] using the eigenvectors of the image Hessian matrix. The Hessian of the intensity image at each point was obtained as a set of values by convolving the image intensity with derivatives of a Gaussian kernel at a fixed scale. The resulting eigenvalues of the Hessian matrix were used to find the corresponding eigenvectors. For a point inside the vessel, the orientations of eigenvectors corresponding to small eigenvalues show the direction along the vessels, while the orientations of the ones relating to large eigenvalues represent for the normal direction. However, for a pixel outside the vessel area this trend is opposite. The locations with a maximum change in orientation of the eigenvectors vector sum represent the maximum change in the contrast of equivalent edge pixels providing a good feature for differentiating vessels from nonvessel pixels at the boundaries. To create the vesselness map, the sum of the two vectors was calculated and the orientation of the resultant vector sum was obtained for each pixel and saved as the orientation matrix. The gradient magnitude of the orientation matrix provided the vesselness map image (Figure 2(b)). 

The gradient magnitude of the orientation matrix transforms the grayscale image with uneven illuminations to a new grayscale image that highlights the vessel edges without the uneven illumination distracter. However, this process adds noise to the background which has to be removed for clear observation of pulsatile motions. To eliminate the background noise, a binary mask was created to separate retinal vasculature from the background pattern on the vesselness map, using two-dimensional Gabor wavelet segmentation with supervised classification method proposed by Soares et al. [21] which is generally the most accepted technique for vessel segmentation [22]. This technique classifies the pixels as vessels or nonvessels by applying 2D Gabor filter at multiple scales and orientations to the gray scale image, for orientations spanning from 0° to 170° with steps of 10°. The maximum modulus of the transform was taken at each scale and over all angles as the response of interest in that specific scale. The rest of the parameters including filter elongation and frequency vector of the complex exponential were set based on the literature (Soares et al. [21]). A supervised classification based on the Bayes decision rule was applied to gain the final segmented image in binary format with white and black pixels representing for vessels and nonvessel areas, respectively (Figure 2(c)).

Prior to the application of the mask, it was corrected for any discontinuities and noise, generated due to the segmentation process. A set of morphological operations was applied to the segmented image for removing the noise and isolated pixels from the background and vessel enlargement. The first step was to consider isolated 1's as speckle and remove any individual 1's surrounded by 0's because an edge always has adjoining transition points. The next step was the generation of the mask for five consecutive dilations with a 3 × 3 structuring element matrix to improve the visibility of the vessels (Figure 2(d)). The details of these morphological operations are available in González and Woods [23]. The vesselness maps were multiplied into the mask to generate the final image set (Figure 2(e)).

### 2.6. Image Sequence Generation

**Figure 3:**
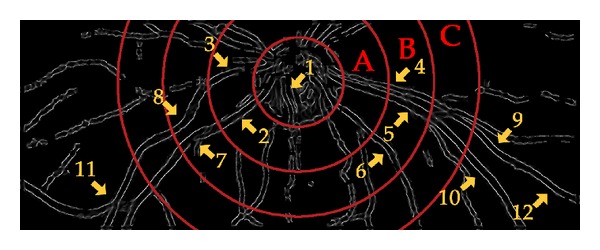
A snapshot from the rendered video for subject no. 11 with concentric circles centered at the optic disk. The arrows point to 12 locations where the pulsation was clearly visible.

To visualize the pulsatile changes, a video for each subject was generated from the masked images obtained in the previous section. A mask of concentric circles was created for each video with the smallest one matching the OD boundary and applied to the video while being played back. The circles divided the regions around the OD into four zones: A, B, C, and greater than C with respect to the OD diameter. The radius of the outer bounding circle was increased by half the OD diameter to obtain the next zone, based on the definitions available in the literature [24] as shown in Figure 3.

The eight images of each subject were placed serially and displayed for 125 ms and rendered to match the standard 30 frames per second format for optimal real-time playback. These were looped fifty times to obtain approximately one-minute playback video for comparing the VMRS with PCA method [8].

## 3. Validation of the Proposed Methodology

The VMRS technique was validated by comparing it against the current state of the art, PCA method [8] in terms of the number of observed pulsatile features in the region of interest (ROI) and qualitative assessment by two independent graders. Two independent grading experts examined all the videos and counted the number of segments in each zone showing pulsatile properties. To compare the VMRS technique with the PCA method, the seven-region-based features defined by Moret et al. [8] were measured by the graders. These features are: spontaneous venous pulsations (SVPs) inside the OD and in zone A, venous pulsation in zone B (VP_B), venous pulsation in zone C and beyond (VP_C), arteriolar pulsation in zone A (AP_A), arteriolar pulsation in zone B (AP_B), arteriolar pulsation in zone C and beyond (AP_C), and serpentine movements and mechanical coupling between the vessels (SM). The graders were also asked to mark each video on a scale of ten with “10” corresponding to best quality and “1” to the poorest and their inability to observe any pulsation in the video due to quality.

### 3.1. Measurement of Individual Vessel Diameter Change for Trend Estimation

**Figure 4:**
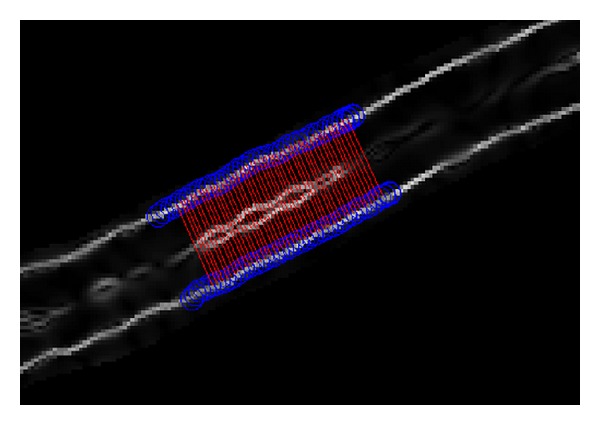
Boundary tracking of a sample vessel segment using the masked vesselness image with highlighted boundaries. The blue circles are the tracked boundaries and the red lines are the shortest Euclidean distance between the two boundaries. The average lengths of these lines are considered as the diameter of the vessel segment on the selected ROI.

A program was written in MATLAB (The Matworks, Inc., R2009a) to measure the vessel diameter and measure the change in the diameter corresponding to pulsatile changes using the vesselness image sequence. In the first frame of the recording, a seed point was identified by a grader for vessel edge tracking purpose, similar to the process used by DVA. The grader specified an ROI on the vesselness image map (Figure 2(e)) by clicking one point around the approximate location of each vessel boundary and specifying the start and end points of the segment that was to be examined. Subsequently, the software automatically tracked the vessel edges using a set of moving circles centered on the edges of the vessels [25, 26]. See Aliahmad et al. [26] for more details about the tracking algorithm. Once the edges were identified, the points on the tracked boundaries corresponding to the two edges of the vessels were paired based on the shortest Euclidean distance (Figure 4). The length of the segment and number of cross-sections were grader selectable, ranging from single point cross-section diameter measurement to measuring over the entire observable length. The diameter of the vessel was defined as the average length of the cross-sectional lines [27–29] within the segment. The above process was repeated for all the image sequences while maintaining the seed point for the segment. The above process was repeated for different vessel segments.

To determine the vessel diameters in micrometer (*μ*m) and adjust the measured values for different image resolutions, digital and optical magnifications [30], a calibration factor was computed as “the ratio of micrometers per pixel in a definite length of a digitized image of the eye fundus” [30]. The distance between the OD center and macula center was considered as the fixed length and was assumed to be 4500 *μ*m which is equivalent to 2.5 times the average OD diameter (1800 *μ*m) reported by Jonas et al. [31] and Pakter et al. [30]. The average distance between the OD center and macula center was obtained based on two expert graders and was found to be 569 pixels averaged over the entire database. Therefore, the calibration factor was obtained as the ratio of 4500/569.1 = 7.90 *μ*m/pixel. 

In the first experiment, only the first frame was demonstrated to the grader and the diameter was measured without seeing the pulsating vessels. In the second experiment, the grader used the video to locate the vessels that were pulsating prior to the caliber measurement. In each experiment, the trends for 35 arterioles and 35 venules were obtained among all the 12 subjects and averaged at each cardiac cycle point to gain a single waveform corresponding to the vessel type. However, there is a potential location-based phase shift between different measuring sites on retinal vessels with respect to OD [32] which would affect the summary diameter estimation. To remove the effect of the difference in the phase delay due to the distance from the OD, the estimated intersubject and intrasubject trends were aligned together using Dynamic Time Warp (DTW) analysis [33], a widely accepted technique for obtaining an optimal alignment between two time dependant sequences. The details of this process is provided in a book by Ao [33].

### 3.2. Statistical Analysis

Statistical analyses were performed using Minitab 16 (Minitab Inc.). For each measurement, the mean diameters across the cardiac points were compared and the overall *P* value of the variations was obtained using repeated measures of ANOVA analysis with post hoc multiple comparison tests. Two different sets of variations were calculated. In the first set, the average diameter at the first cardiac point was considered as the reference and the absolute value of the deviations from that point was found at other cardiac point. In the second set, the difference between the maximum and the minimum diameters was calculated to study the maximum change across a cardiac cycle with respect to the vessel type and the measurement method. 

## 4. Results

Table 1 shows a comparison of the visualization using PCA [8] and the proposed technique. Column 1 corresponds to the video numbers (subjects). The number of pulsating segments observed in each zone by the graders is presented in columns 2 to 31 and columns 32–35 show the scale-ten quality index for both techniques as reported by two independent graders. From this table, it is observed that there is a significant increase in the average number of pulsatile segments when the visualization was performed using VMRS compared with PCA. The results also show that there is a significant improvement in the mean quality index, from 3 to 6.9.

Figure 3 shows the location of the different features in the detected pulsations in each zone for subject no. 11. The following features at each numbered location have been marked: (1) SVP inside the optic disk, (2) vein diameter variation along the trunk or SVP if defining it within one OD diameter region, (3) arterial pulsation in zone A, (4) arterial pulsation in zone B, (5) vein diameter variation in zone B, (6) fine vessel pulsation, (7) and (8) serpentine movements, mechanical coupling between the vessels and pulsation, and (9) to (12) diameter variation outside the zone C. 

The change of individual vessel caliber due to pulsation, measured without VMRS visualization, has been shown in Table 2. The results show that the average change in vessel diameter during the cardiac cycle was 1.2 *μ*m for arteries and 1.6 *μ*m for the veins. The statistical analysis showed no significance for the variations across cardiac cycle for both vessel types (all *P* values for variations >0.05). 

The change of the vessel diameter over the cardiac cycle after visualization, averaged for 12 subjects, has been tabulated in Table 3. The results show 3.3 *μ*m change for arterioles and 6.6 *μ*m change for the venules. There is a significant increase from the measurements without visualization (all *P* values for variations <0.01).

**Figure 5:**
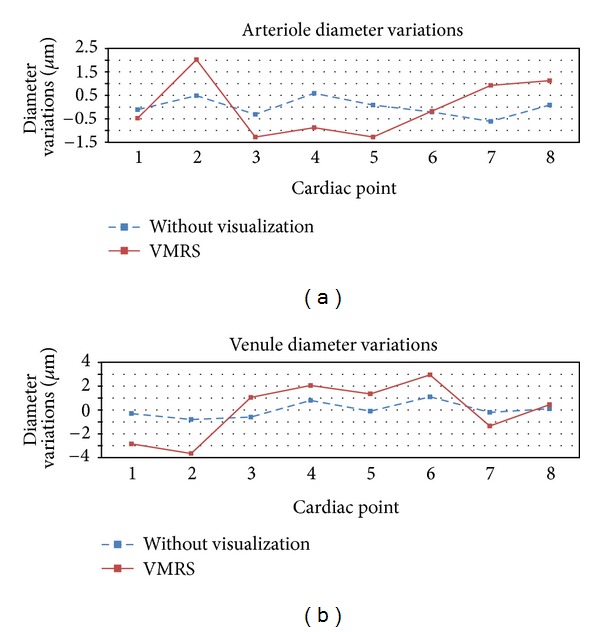
Variation of individual vessel diameter at different cardiac points. (a) Average arteriole caliber change across one cardiac cycle and (b) average venule caliber change across one cardiac cycle.

Figure 5 shows the trend for vessel diameter variation over the cardiac cycle. All the trends for both arterioles and venules followed approximately a similar pattern which was in agreement with the result from previous works [7, 8]. However, the result from VMRS (after visualization) showed higher variations than the other test especially for the venules. Arteries and veins were found to be out of phase with maximum peaks at different cardiac points. Arterioles peaked at 1/8th of the R-R interval (point no. 2), but the venules did at its 5/8th (point no. 6). 

## 5. Discussion

In this study, we have presented and validated the VMRS technique for visualization of pulsatile properties of retinal vasculature with respect to cardiac cycle. This method enables the visualization of pulsatile movements of the retinal vasculature using a commercially available standard non-mydriatic fundus camera with minimum modification of ECG synchronization. The results indicate that when this method was used, the graders were able to better observe the pulsation of the retinal vasculature, and judged the quality of the video to be better than when using PCA-based technique. The results also show that VMRS is able to identify more numbers of vessels compared with PCA, indicating greater sensitivity of the proposed method. Another advantage of VMRS is that the video shows better contrast which makes the grading of the retinal images easier for the graders. 

There are number of pulsatile features such as SVP, serpentine movements, vessel displacement, and mechanical coupling that can be visible on a single-vessel segment. The results of this work, and other papers [8] have shown that the vessel pulsations may only be visible and measureable on a certain vessel segment. This indicates that it is important to identify the location of pulsation prior to obtaining the measurements. VMRS provides a method using which the graders can locate the suitable pulsating segments and conduct their measurements on these segments. 

Another advantage of VMRS is that it allows the graders to view greater number of regions simultaneously. While PCA method crops the images around OD to include the primary vessels due to the normalization process (subtracting average of the whole sequence from each image) which performs better in a small region; VMRS does not limit the grading region to a specific distance from the OD (Figure 3). This advantage provides the ability of examining the pulsatile properties across a longer segment of major retinal vessels including the branches. This method is expected to provide a simple and effective application in retinal pulse wave velocity (PWV) measurement [34] using fundus imaging.

The outcome of this work has demonstrated and validated a new method that can be used to monitor the pulsation of retinal vasculature. The next step is to determine the clinical efficacy of this method for early identification of disease and PWV measurements. The authors are also interested in identifying the possible reasons for the difference in the location of the pulsatile vessels among different healthy subjects as observed in this study.

## Figures and Tables

**Table 1:** Comparison between the PCA and the proposed method. Columns 2–31 are the number of pulsative segments observed by the graders, while columns 32–35 are the scale-ten quality measures.

Subject no.	Pulsatile features																															
	SVP^a^				VP_B^b^				VP_C^c^				AP_A^d^				AP_B^e^				AP_C^f^				SM^g^				QOF^h^			
	PCA		Proposed		PCA		Proposed		PCA		Proposed		PCA		Proposed		PCA		Proposed		PCA		Proposed		PCA		Proposed		PCA		Proposed	
			method				method				method				method				method				method				method				method	
	G1	G2	G1	G2	G1	G2	G1	G2	G1	G2	G1	G2	G1	G2	G1	G2	G1	G2	G1	G2	G1	G2	G1	G2	G1	G2	G1	G2	G1	G2	G1	G2
1	3	3	4	4	1	2	3	4	1	1	3	3	0	0	4	5	1	0	3	3	0	0	3	2	2	1	3	3	4	3	8	7
2	3	3	3	3	2	1	3	3	0	0	3	3	2	2	3	4	1	0	4	4	0	1	4	4	1	1	3	3	3	4	7	8
3	1	1	3	3	2	0	3	3	1	3	2	3	1	2	3	3	1	2	2	2	0	0	1	1	1	1	1	1	2	4	7	7
4	3	3	4	5	3	2	3	3	1	2	2	2	1	1	2	2	0	0	1	2	0	0	1	1	0	0	1	1	3	2	6	7
5	1	1	1	1	0	1	0	0	3	4	2	2	1	1	0	0	2	3	1	1	0	1	0	0	2	2	1	1	4	1	6	6
6	3	3	2	2	2	2	1	1	2	2	2	2	2	3	3	3	2	2	2	2	1	1	2	2	2	2	2	2	4	4	8	7
7	1	2	1	1	2	3	1	1	1	3	1	2	0	0	1	1	0	1	2	2	0	1	0	0	2	2	2	2	1	3	7	6
8	3	3	3	3	2	2	3	2	1	2	2	2	1	1	1	1	0	1	1	2	0	0	1	1	0	0	0	0	2	3	6	7
9	3	3	4	4	1	1	3	2	1	1	3	2	1	1	1	1	0	0	2	1	0	0	1	0	1	1	2	2	2	2	6	6
10	3	2	3	2	3	2	3	2	2	2	2	2	1	2	1	1	1	2	1	1	0	2	1	1	1	1	1	1	3	2	8	6
11	3	3	2	2	1	1	2	1	2	3	1	2	2	2	1	2	3	4	3	2	2	3	3	3	2	2	2	2	6	5	9	9
12	1	2	1	1	1	2	0	0	0	0	0	0	1	1	1	1	0	0	0	0	0	0	0	0	2	2	1	1	2	2	5	6

Average	2.3	2.4	2.6	2.6	1.7	1.6	2.1	1.8	1.3	1.9	1.9	2.1	1.1	1.3	1.8	2.0	0.9	1.3	1.8	1.8	0.3	0.8	1.4	1.3	1.3	1.3	1.6	1.6	3.0	2.9	6.9	6.8

Average of two graders	2.4		2.6		1.6		2.0		1.6		2.0		1.2		1.9		1.1		1.8		0.5		1.3		1.3		1.6		3.0		6.9	

^a^Spontaneous venous pulsation within one OD diameter from the center; ^b^venous pulsation in zone B; ^c^venous pulsation in zone C and beyond; ^d^arterial pulsation in zone A; ^e^arterial pulsation in zone B; ^f^arterial pulsation in zone C and beyond; ^g^serpentine movements and mechanical coupling between arteries and veins; and ^h^quality of the observed features.

**Table 2:** Average pulsation of retinal vasculature without visualization at different cardiac cycle points for 12 subjects.

	Cardiac cycle point	Mean (SD) (*μ*m)	*P* value^†^	Variations* (*μ*m)	Max–Min (*μ*m)
Individual arterioles caliber	1	90.6 (10.0)		Ref.	
	2	91.2 (9.8)		0.6	
	3	90.4 (10.3)		0.2	
	4	91.3 (9.4)	0.699	0.7	1.2
	5	90.8 (9.1)		0.2	
	6	90.5 (8.7)		0.1	
	7	90.1 (9.7)		0.5	
	8	90.8 (9.9)		0.2	

Individual venules caliber	1	111.9 (12.6)		Ref.	
	2	111.4 (11.7)		0.5	
	3	111.6 (13.1)		0.3	
	4	113.0 (13.2)	0.211	1.1	1.9
	5	112.1 (12.9)		0.2	
	6	113.3 (12.5)		1.4	
	7	112.0 (14.3)		0.1	
	8	112.3 (13.6)		0.4	

*Absolute value compared to cycle point 1; ^†^overall *P* value for variations.

**Table 3:** Average pulsation of retinal vasculature with visualization at different cardiac cycle points for 12 subjects.

	Cardiac cycle point	Mean (SD) (*μ*m)	*P* value^†^	Variations* (*μ*m)	Max–Min^†^ (*μ*m)
Individual arterioles caliber	1	99.0 (9.5)		Ref.	
	2	101.5 (10.0)		2.5	
	3	98.2 (9.6)		0.8	
	4	98.6 (9.7)	0.001	0.4	3.3
	5	98.2 (9.8)		0.8	
	6	99.3 (11.1)		0.3	
	7	100.4 (9.7)		1.4	
	8	100.6 (8.4)		1.6	

Individual venules caliber	1	109.4 (14.5)		Ref.	
	2	108.6 (15.0)		0.8	
	3	113.3 (16.1)		3.9	
	4	114.3 (14.5)	<0.001	4.9	6.6
	5	113.6 (17.3)		4.2	
	6	115.2 (15.7)		5.8	
	7	110.9 (16.1)		1.5	
	8	112.7 (17.2)		3.3	

*Absolute value compared to cycle point 1; ^†^overall *P* value for variations.
